# Effect of Childhood Pneumococcal Conjugate Vaccination on Invasive Disease Serotypes in Serbia

**DOI:** 10.3390/vaccines12080940

**Published:** 2024-08-22

**Authors:** Nataša Opavski, Miloš Jovićević, Jovana Kabić, Dušan Kekić, Ina Gajić, Study Group for Laboratory Surveillance of Invasive Pneumococcal Diseases

**Affiliations:** Institute of Microbiology and Immunology, Faculty of Medicine, University of Belgrade, 11000 Belgrade, Serbia; jovicevic.milos@gmail.com (M.J.); dusan_vk@yahoo.com (D.K.)

**Keywords:** *Streptococcus pneumoniae*, PCV, invasive pneumococcal disease, IPD, serotypes, sequence types, Serbia, resistance

## Abstract

In Serbia, PCV10 was introduced into the routine immunization for children under 2 in 2018 and replaced by PCV13 in 2022. We evaluated their impact on the distribution of invasive pneumococcal disease (IPD) serotypes across all age groups. Overall, 756 isolates were obtained from patients with IPD between 2010 and 2023 through laboratory surveillance. In the post-vaccination period, serotypes 14, 19F, 23F, and 6A significantly declined, while 3 and 19A considerably increased. This was especially evident in the ≤2 years group, making these serotypes the most prevalent among them. Serotype 3 dominated, representing 19.1% of all invasive isolates prior to 2018 and 33.1% thereafter. While serotype coverage of PCV10 has significantly decreased in the ≤2 years group (from 74.2% before 2018 to 29.5% after 2018), PCV13 coverage was 63.9% after 2018. In the post-PCV period, non-PCV13 serotypes, such as 9N, 10A, 15A, 15B, 15C, 22F, 6C, 6D, and 7C, increased across all isolates. Antibiotic non-susceptibility considerably decreased after 2018. MLST analysis showed shifts in sequence type prevalence, with pre-PCV lineages replaced and ongoing serotype 3 persistence, alongside potential capsule-switching events. These findings emphasize a noticeable shift in the distribution of serotypes and adaptability of pneumococcal populations, highlighting the importance of ongoing surveillance and the requirement for the urgent introduction of higher valent vaccines into the National Immunization Program.

## 1. Introduction

*Streptococcus pneumoniae* is a leading cause of both non-invasive diseases (such as acute otitis media, sinusitis, and non-bacteremic pneumonia) and invasive diseases (including bacteremic pneumonia, sepsis, and meningitis, collectively known as invasive pneumococcal diseases, or IPDs) across all age groups. It was confirmed that pneumococcal pneumonia is the leading cause of lower respiratory infection morbidity and mortality globally and caused more deaths than all other etiologies combined [1]. The most susceptible population groups are children younger than 5 years and adults older than 65 years. In 2017, the World Health Organization (WHO) classified penicillin-non-susceptible *S. pneumoniae* as a medium-priority pathogen on the WHO Bacterial Priority Pathogens List. By 2024, the WHO updated its list to include macrolide-resistant pneumococci in the same medium-priority category [2].

For the prevention of pneumococcal diseases, polysaccharide pneumococcal 23-valent vaccine (PPV23) and particularly pneumococcal conjugated vaccines (PCVs) play pivotal roles. In developed countries, a comprehensive vaccination schedule that includes both conjugate and polysaccharide vaccines is implemented to protect at-risk populations of all age groups, while the United States strongly advises routine administration of pneumococcal conjugate vaccine for all adults older than 65 years [3]. PCVs have been in use for over two decades, with a steady expansion in their global reach and coverage.

Currently, four PCVs are actively utilized. PCV15 and PCV20 have only recently become accessible, although PCV10 and PCV13 have been in use for roughly 15 years. The widespread administration of conjugate vaccines among infants has notably decreased the incidence of IPD, as well as pneumonia and otitis media [4,5,6]. In addition to the impact on IPD rates, PCV introduction was also followed by a reduction in antibiotic resistance rate [7].

Since these vaccines provide herd protection, their effect is observed not only in the vaccinated group but also in unvaccinated older children and adults in populations with >70–80% vaccine coverage [8,9]. However, a decrease in vaccine type (VT) carriage is frequently linked with a rise in non-vaccine type (NVT) carriage but also illnesses attributable to NVT [5].

Considering the unique characteristics of each country, it is strongly recommended that every country conducts surveillance on serotype distribution prior to vaccine introduction, with particular emphasis on monitoring serotype changes in the subsequent years. The obtained data are expected to provide crucial information for immunization policy and can serve as a valuable model for countries implementing routine PCV vaccination across diverse settings [10].

Although reporting of IPD is mandatory in Serbia, its implementation in practice is inadequate, and there is a lack of relevant data on the IPD rate. In addition, during the COVID-19 pandemic in 2020 and 2021, a small number of IPD cases were reported. Therefore, the impact of PCV on the incidence of IPD could not be monitored. Nevertheless, the National Reference Laboratory (NRL) for Streptococci conducts national passive voluntary laboratory-based surveillance on IPD and serotype distribution. This enables us to monitor changes in serotypes and resistance profiles within the pneumococcal population following the introduction of PCVs in our country.

In Serbia, the mandatory application of PCV for children less than 2 years of age began in 2018, with the administration of PCV10 using schedule 3 + 1. This was replaced by PCV13 in 2022 with the same schedule and then switched to the 2 + 1 program in 2023. From 2024, PCV10 is once again being used applying the 2 + 1 schedule. While the immunization coverage of children under 2 years has been high (>90% in the period 2019–2023) [11], the compliance rate of PCVs/PPV23 among older children and adults is notably low, despite its requirement for numerous at-risk groups as outlined in the rulebook [12].

In countries where PCVs have been used in the childhood immunization program for at least eight years, it has been noted that both PCV13 and PCV10 programs appear to reduce IPD incidence overall. However, the distribution of serotypes varied depending on which PCV was being used [13].

To assess the impact of PCVs, this study investigated serotype and clonal distribution, as well as antimicrobial resistance among pneumococcal invasive isolates before and after PCV introduction in Serbia. The focus was on emerging serotypes and clones in the post-PCV era in Serbia, aiming to provide updated information for future vaccine strategies.

## 2. Materials and Methods

### 2.1. Bacterial Collection, Identification, and Conservation of Isolates

The study was carried out as a laboratory-based retrospective and prospective study on invasive pneumococcal isolates. The retrospective study covered the period between 1 January 2010 and 31 December 2017 (pre-vaccination period), while the prospective study included the period from 1 January 2018 to 31 December 2023 (post-vaccination period). Altogether, the study covers a period of 14 years. All invasive pneumococcal isolates and the clinical and demographic data received in the NRL for streptococci at the Medical Faculty University of Belgrade, Serbia, during this period were included in the study. During this period, isolates were sent from a total of 31 laboratories situated in secondary and tertiary healthcare centers, collectively serving over 70% of the country’s population. A case of IPD was defined as isolation of *S. pneumoniae* from a normally sterile body site (blood, cerebrospinal fluid (CSF), pleural, peritoneal, and joint fluid).

Pneumococcal isolates from clinical microbiological laboratories were initially identified by conventional bacteriological methods (colony’s morphology, α-hemolysis, Gram staining, agglutination tests, optochin susceptibility, bile solubility) or using an automated VITEK^®^2 system (bioMérieux, Marcyl’Étoile, France).

Before 2020, the confirmation of *S. pneumoniae* by the NRL for streptococci was performed via PCR detection of the *lytA* gene [14] (Gillespie et al., 1994), and after 2020, it was conducted using MALDI-TOF mass spectrometry (VITEK-MS) (bioMérieux, Marcyl’Étoile, France). All isolates were stored at −70 °C in the skim milk, tryptone, glucose, glycerol (STGG) medium for further analysis.

Patients were divided into five age groups: ≤2 years; >2–≤ 5 years; >5–≤18 years; >18–<65 years; and ≥65 years.

### 2.2. Serotyping and Antimicrobial Susceptibility Testing

The serotyping of *S. pneumoniae* isolates was performed via Quellung reaction, using the chessboard system and specific sera (Statens Serum Institute, Copenhagen, Denmark).

Antimicrobial susceptibility testing (AST) was performed using the disk diffusion method for oxacillin, erythromycin, clindamycin, norfloxacin, tetracycline, chloramphenicol, trimethoprim-sulfamethoxazole, and vancomycin (Bio-Rad, Watford, UK) [15]. Macrolide resistance phenotypes were determined using a double disk diffusion test with erythromycin (15 μg) and clindamycin (2 μg) disks. The MIC Test Strip determined the minimum inhibitory concentrations (MICs) of penicillin, ceftriaxone, and erythromycin (Liofilchem, Roseto degli Abruzzi, Italy). All AST results were interpreted according to the European Committee on Antimicrobial Susceptibility Testing (EUCAST, 2023) guidelines [16]. *S. pneumoniae* ATCC 49619 was used as the control strain.

Isolates exhibiting MICs > 0.06 mg/L for penicillin and >0.5 mg/mL for ceftriaxone were categorized as penicillin/ceftriaxone non-susceptible *S. pneumoniae* (PNSP, CNSP) strains due to their expression of varying degrees of resistance [17]. Also, all isolates identified as I or R for macrolides, fluoroquinolones, and trimethoprim-sulfamethoxazole were classified as non-susceptible (NS) [18]. Furthermore, strains that were non-susceptible/resistant to three or more antibiotic classes were defined as multidrug-resistant (MDR). Accordingly, isolates that were non-susceptible/resistant to five or more classes of antibiotics were defined as extensive drug resistance (XDR) [19].

### 2.3. Multilocus Sequence Typing

Multilocus sequence typing (MLST) was performed on a total of 246 isolates of *S. pneumoniae*, with 172 isolates originating from the pre-vaccination period and 74 from the post-vaccination period. Isolates were randomly chosen from all participating institutions to determine the serotype frequency, relevance, and penicillin and macrolide resistance levels. The MLST of the seven housekeeping genes (*aroE*, *gdh*, *gki*, *recP*, *spi*, *xpt*, and *ddl*) was performed following previously reported techniques [20]. Sequence types (STs) were assigned based on the allelic patterns of these genes using the MLST database (http://pubmlst.org/spneumoniae/, accessed on 1 May 2024). In cases of new alleles or allelic profiles, data were submitted to the database curator for the assignment of ST numbers. In addition, the geoBURST technique was used to construct a minimum spanning tree indicating ST connections, which was then displayed using the PHYLOVIZ program (vesion number 2.0) [21]. STs that shared at least five of the seven allelic variations were placed in the same clonal complex (CC) [22]. The resulting STs were then matched to Pneumococcal Molecular Epidemiology Network (PMEN) clones to discover worldwide antibiotic-resistant clones.

### 2.4. Statistical Analysis

Statistical analysis was performed using SPSS version 20.0 (SPSS Inc., Chicago, IL, USA). To compare categorical variables, the Pearson chi-squared test or Fisher exact test were used. *p*-values of <0.05 were considered statistically significant.

## 3. Results

### 3.1. Bacterial Isolates and Patients’ Characteristics

**Figure 1:**
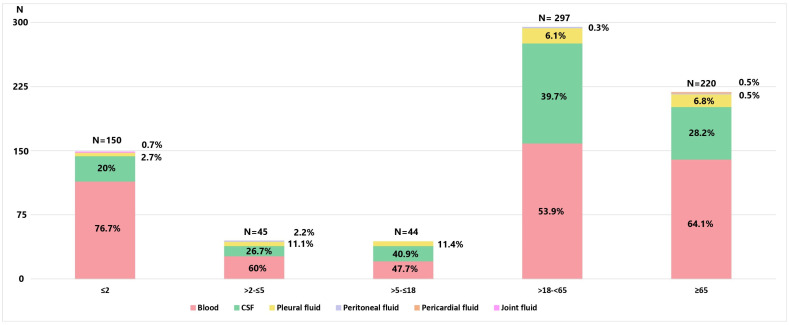
Distribution of patients with IPD by age groups and sources from which *S. pneumoniae* was isolated.

During 2010–2023, a total of 756 invasive non-duplicate *S. pneumoniae* isolates were collected: 424 in the pre-vaccination period and 332 in the post-vaccination period. The number of isolates per year was 53 in 2010; 24 in 2011; 31 in 2012; 61 in 2013; 62 in 2014; 49 in 2015; 75 in 2016; 69 in 2017; 64 in 2018; 64 in 2019; 29 in 2020; 20 in 2021; 73 in 2022; and 82 in 2023. Based on the isolates we received, it could be estimated that notification rates varied over the years, with the lowest rates observed during COVID-19 years (0.30 cases per 100,000 population). In the years following COVID-19, when the annual number of isolates stabilized, the notification rates were 1.09 cases per 100,000 population in 2022 and increased to 1.23 cases per 100,000 population in 2023. Isolates were obtained from 435 (57.5%) males with a median age of 55.5 years (2 months–90 years) and 321 (42.5%) females with a median age of 51 years (3 months–92 years). The distribution of isolates across different age categories, as well as the specimen from which they were isolated, are presented in Figure 1. The majority of strains were isolated from adult patients (517; 86.4%), with 195 (25.8%) coming from children under 5 years of age. Isolates mostly originated from blood (464; 61.4%), CSF (240; 31.7%), and pleural fluid (47; 6.2%). Overall, occult bacteremia and septicemia were reported in 414 (54.8%), meningitis in 240 (31.8%), bacteremic pneumonia in 97 (12.8%), empyema pleurae in 30 (6.1%), peritonitis in 3 (0.4%), and pericarditis and arthritis in 2 (0.2%) cases.

### 3.2. Serotype Distribution and Serotype Vaccine Coverage Over the Two Study Periods

**Figure 2:**
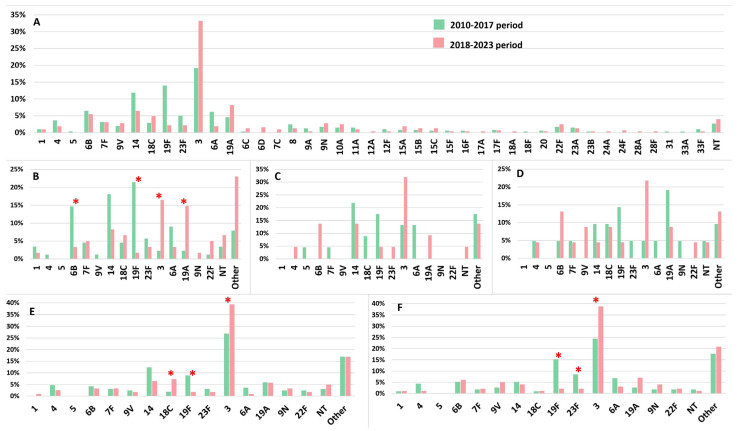
Distribution of serotypes by age group among invasive *Streptococcus pneumoniae* isolates in the pre-vaccine (2010–2017) and post-vaccine (2018–2023) period. * *p* < 0.05. (**A**) All age groups; (**B**) ≤2 years; (**C**) >2–≤5 years; (**D**) >5–≤18 years; (**E**) 18–<65 years; (**F**) ≥65 years. NT—non-typable. (**B**) Other serotypes in the post-PCV period, declining in order: 15A, 6D, 24F, 6C, 7C, 12A, 15B, 18A, 20, and 23A. (**C**) Other serotypes in the post-PCV period, declining in order: 6D, 8, and 23A. (**D**) Other serotypes in the post-PCV period, declining in order: 15A, 23A, and 28A. (**E**) Other serotypes in the post-PCV period, declining in order: 10A, 15C, 7C, 6C, 6D, 8, 15A, 15B, 15F, 16F, 17A, 23A, 23B, and 24A. (**F**) Other serotypes in the post-PCV period, declining in order: 10A, 11A, 6C, 8, 15B, 17F, 6D, 9A, 12F, 15A, 28F, and 33F.

The serotype distribution across all age groups, including specific age groups (only for serotypes represented by more than 2%), is provided for both the pre-vaccination (2010–2017) and post-vaccination (2018–2023) periods, as depicted in Figure 2. Overall, 44 IPD-associated serotypes were identified, with 34 in the pre-PCV period and 40 in the post-PCV period. The most common pneumococcal serotypes in the period before 2018 were 3 (19.1%), 19F (13.9%), 14 (11.8%), 6B (6.4%), and 6A (6.1%), accounting for 57.3% of all isolates (Figure 2A). In the period after 2018, serotype 3 dominated, being responsible for 33.1% of all IPD cases, followed by types 19A (8.1%), 14 (6.3%), and 6B (5.4%). Statistically significant (*p* < 0.05) decreases in the prevalence of serotypes 14, 19F, and 23F, covered by both PCV10 and PCV13 vaccines, as well as serotype 6A, covered only by PCV13, were observed in all IPD cases when comparing the periods before and after the introduction of vaccination (Figure 2A).

When analyzed by subgroups, children under 2 years old showed a significant decrease in serotypes 6B and 19F. In the 18–64 age group, there was a significant reduction in serotype 19F, while in the 65 and older age group, serotypes 19F and 23F showed statistically significant decreases (Figure 2B,E,F). However, despite the decrease in the prevalence of serotype 14, it remains one of the leading causes of IPD in the post-PCV period. There was a statistically significant (*p* < 0.05) increase in serotype 3 among children ≤2 years old and adults. The considerable increase (*p* < 0.05) in the prevalence of serotype 19A was particularly pronounced in the youngest pediatric group (≤2 years) (Figure 2B). No statistically significant change was detected between the two periods among other PCV10/13 serotypes.

Altogether, in the post-PCV period, among children younger than 5 years, serotype 3 was dominant, followed by serotypes (in decreasing frequency) 19A, 14, 18C, 7F, and 23F in the ≤2 years group and 6B, 14, and 19A in the >2–≤5 years group. Among adults, in addition to serotype 3, which predominated overall, serotypes 18C, 14, and 19A were more common in the >18–<65 age group, while serotypes 19A, 6B, 9V, and 14 remained significant causes of IPD in those aged ≥65 years.

**Figure 3:**
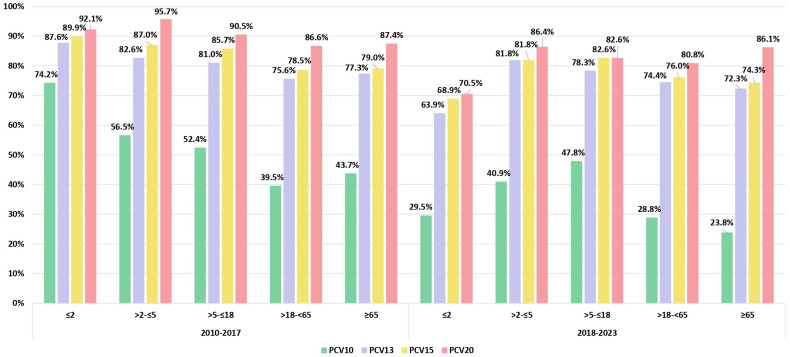
The serotype coverage of PCV10, PCV13, PCV15, and PCV20 is shown according to the observed proportion of the serotypes included in each of these vaccines for both study periods (pre-PCV and post-PCV) and stratified by age groups (≤2; >2–≤5; >5–<18; >18–<65; and ≥65).

Figure 3 illustrates the coverage of the PCV10, PCV13, PCV15, and PCV20 based on the observed proportions of the serotypes included in each of these vaccines for both study periods and stratified by age groups. The most significant decline in PCV serotype vaccine coverage was observed for PCV10 in the group of children under 2 years of age, dropping from 74.2% before 2018 to 29.5% after 2018 (*p* < 0.05). In the same group, PCV13 serotype coverage decreased from 87.6% to 63.9%, while potential serotype coverage for PCV15 and PCV20 in the post-PCV period would be 68.9% and 70.5%, respectively. In the group of children >2–≤5 years, the PCV10 serotype coverage dropped from 56.5% to 40.9% (*p* = 0.295). Meanwhile, PCV13, PCV15, and PCV20 exhibited a much smaller decline in serotype coverage during the same period. In other age groups, the serotype coverage of PCV13, PCV15, and PCV20 did not significantly decrease in the post-PCV period, maintaining a relatively stable level. In adults over 18, PPV23 serotype coverage remained stable throughout the period, keeping a consistent level of 86%.

When analyzing the increase in the incidence of non-PCV13 serotypes in both the before and after vaccination periods, it is evident that the rise is most pronounced and statistically significant (*p* < 0.05) in children under 2 years of age, rising from 12.4% before 2018 to 36.1% after 2018. In other age groups, the increase is less noticeable: in >2–≤5 from 17.4% to 18.2%; in >5–≤18 from 19.0% to 21.7%; in >18–<65 from 24.4% to 25.6%; and in ≥65 from 22.7% to 27.7%.

In the post-PCV period, non-PCV13 serotypes, such as 9N, 10A, 15A, 15B, 15C, 22F, 6C, 6D, and 7C, increased across all isolates, though none of these changes were statistically significant. Among children under 2 years old, there were 14 non-PCV13 serotypes, with 6D, 15A, 22F, and 24F being the most common. In the >2–≤ 5 age group, serotypes 6D and 8 showed an increase, while in the >5–≤18 age group, serotypes 15A, 22F, 23A, and 28A rose. For adults aged >18–<65, there was a rise in serotypes 7C, 9N, 10A, and 15C. In adults over 65 years old, an increase in non-PCV13 serotypes 6C, 9N, 10A, 11A, 15B, 17F, and 22F was observed.

Serotypes 3, 18C, and 19A were significantly more frequently isolated from meningitis cases than other serotypes (*p* < 0.05). Also, serotypes 3 and 18C were more frequent causes of bacteremic pneumonia cases than other types (*p* < 0.05).

### 3.3. Antimicrobial Susceptibility

**Figure 4:**
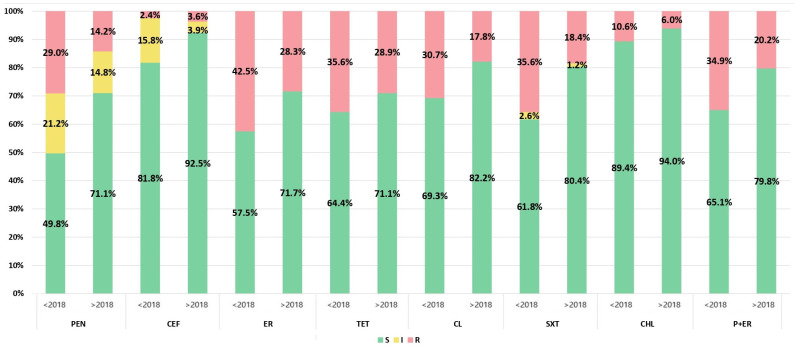
Antimicrobial susceptibility of invasive pneumococcal isolates in the pre-vaccination and post-vaccination periods. Left chart bar <2018: 2010–2017 period; right char bar >2018: 2018–2023 period. S (susceptible), I (susceptible, increased exposure), and R (resistant) isolates; PEN, penicillin; ER, erythromycin; CEF, ceftriaxone; KL, clindamycin; TET, tetracycline; SXT, trimethoprim-sulfamethoxazole; CHL, chloramphenicol; P+ER, penicillin and erythromycin co-resistance.

After the introduction of PCV, a statistically significant decrease (*p* < 0.05) in antibiotic non-susceptibility was observed among invasive pneumococcal isolates for all antibiotics except tetracyclines, fluoroquinolones, and vancomycin (Figure 4). For example, the percentage of non-susceptibility decreased from 50.2% to 28.9% for penicillin, from 18.2% to 7.5%% for ceftriaxone, and from 42.5% to 28.3% for erythromycin. All isolates were susceptible to vancomycin, while resistance to fluoroquinolones remained very low even before the introduction of PCV (1.2% before 2018 and 0.9% after 2018). Also, the number of isolates with co-resistance to penicillin and erythromycin decreased from 34.9% to 20.2%, while the percentage of MDR and XDR isolates dropped from 21.0% to 16.9% and from 17.5% to 7.8%, respectively. MDR isolates were statistically significantly more frequently isolated from children under 5 years of age (29%) (*p* < 0.05), while XDR isolates were more common in children under 2 years of age (32%) and adults under 65 years of age (27%).

Among macrolide-resistant isolates, the cMLS phenotype constituted 68.3%, the iMLS phenotype accounted for 3.4%, and the M phenotype represented 28.3% of the cases before 2018, whereas in the later period, these values were 61.3%, 2.1%, and 36.6%, respectively.

The serotypes with the highest resistance rates were 6A, 6B, 14, 18C, 19A, 19F, and 23F (Table 1). Serotypes 6B, 14, 19A, and 19F were statistically significantly more resistant to almost all antibiotics except fluoroquinolones and vancomycin compared to other serotypes, and they accounted for the highest number of MDR and XDR isolates. However, in the post-PCV period, the prevalence of most of the serotypes mentioned above decreased, leading to a reduction in the rate of resistance. The most prevalent serotype, 3, was statistically significantly (*p* < 0.05) more susceptible to antibiotics than the other serotypes.

### 3.4. Multilocus Sequence Typing

**Figure 5:**
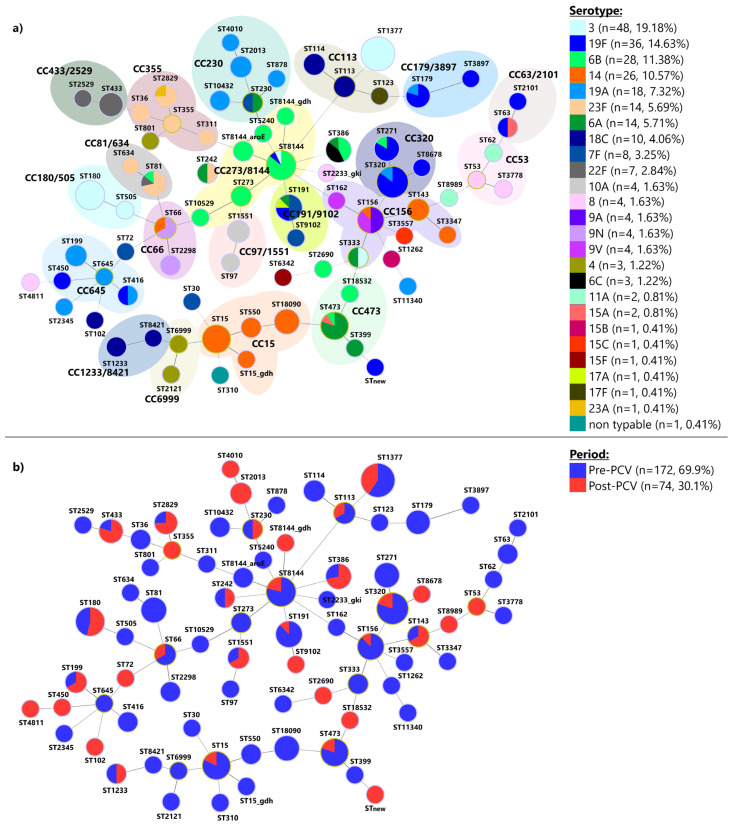
Minimum spanning trees of MLST for 246 invasive *Streptococcus pneumoniae* isolates over the 14-year period: (**a**) Minimum spanning tree representing the detected sequence types (STs) and their corresponding serotypes. Each circle represents a ST, and the circle’s color represents the detected serotype. The area of each circle corresponds to the number of isolates, and pastel zones between some groups of circles indicate that these profiles belong to the same clonal complex (CC); (**b**) Minimum spanning tree representing detected STs regarding the study period in which they were detected.

The MLST analysis of 172 pneumococcal isolates from the pre-PCV period and 74 isolates from the post-PCV period revealed 78 distinct STs distributed among 20 CCs, consisting of 59 STs and 19 singletons (Figure 5a). Among these, 44 STs were identified from a single isolate each, while 34 STs were associated with multiple isolates. The most frequently occurring CCs and STs throughout the entire period were ST1377 (*n* = 32), CC320 (*n* = 27), CC15 (*n* = 20), CC273/8144 (*n* = 20), CC156 (*n* = 14), CC180/505 (*n* = 14), CC473 (*n* = 12), and CC191/9102 (*n* = 9), collectively representing 57.8% (*n* = 148) of all isolates. Within the 78 identified STs, 5 (6.4%) were novel entries in the PubMLST database. Of these, two were present as singletons in our population, and three were incorporated into existing CCs (Figure 5a).

Additionally, potential capsule-switching events were observed in several STs (Figure 5a): ST63 (serotypes 15A and 19F), ST66 (serotypes 9N and 14), ST81 (serotypes 23F, 22F, and 6B), ST156 (serotypes 14, 9V, and 9A), ST179 (serotypes 19A and 19F), ST191 (serotypes 6A, 7F, 17A, and 19F), ST230 (serotypes 6A and 7F), ST242 (serotypes 6A and 23F), ST271 (serotypes 19F and 6B), ST320 (serotypes 19A and 19F), ST333 (serotypes 3 and 6A), ST386 (serotypes 6A, 6B, and 6C), ST416 (serotypes 19A and 19F), ST473 (serotypes 6A, 6B, and 15A), ST2829 (serotypes 23A and 23F), and ST8144 (serotypes 3, 6B, and 19F). Among them, several were detected only after the introduction of PCVs. These include ST66 (from serotype 9N to 14), ST230 (from serotype 7F to 6A), ST242 (from serotype 6A to 23F), ST320 (from serotype 19F to 19A), ST386 (from serotypes 6A and 6C to 6B), and ST2829 (from serotype 23A to 23F) (Supplementary Table S1).

Figure 5b illustrates that certain lineages are unique to either the pre-PCV or post-PCV periods. Specifically, of the 78 STs identified, 44 were exclusive to the pre-PCV period, whereas 15 were unique to the post-PCV period. Only 19 STs were found to persist across both study periods. Within the STs exclusive to the pre-PCV period, 31 were linked to PCV13 serotypes. In contrast, among the STs identified solely in the post-PCV period, 13 were associated with PCV13 serotypes.

A total of 17 international Pneumococcal Molecular Epidemiology Network (PMEN) clones and their single-locus variants (SLVs) or double-locus variants (DLVs) were identified among 43 STs, including Spain^23F^-1, Spain^9V^-3, Tennessee^23F^-4, England^14^-9, Taiwan^19F^-14, Taiwan^23F^-15, Tennessee^14^-18, Portugal^19F^-21, Greece^6B^-22, Sweden^15A^-25, Netherlands^3^-31, Denmark^14^-32, Netherlands^8^-33, Netherlands^18C^-36, Netherlands^15B^-37, Sweden^4^-38, and Netherlands^7F^-39 (Supplementary Table S1). Among them, 13 different international PMEN clones and their SLVs or DLVs were identified both in the pre-PCV and post-PCV periods, while 4 different PMEN clones were detected only before the introduction of the PCVs (Spain^23F^-1, Portugal^19F^-21, Sweden^15A^-25, and Sweden^4^-38) (Supplementary Table S1).

## 4. Discussion

This investigation represents the first comprehensive analysis detailing the shifts in serotype distribution, antimicrobial resistance, and clonal composition of invasive *S. pneumoniae* in Serbia over a 14-year period spanning pre- and post-vaccine periods. The obtained results revealed that six years after the introduction of PCVs into the National Immunization Program (NIP) as mandatory in Serbia, the prevalence of PCV10 and PCV13 serotypes, specifically 14, 19F, 23F, and 6A, has considerably declined in all age groups, leading to a shift in the profile of serotypes causing IPD. A previous nationwide study demonstrated that the most predominant serotypes in Serbia during the pre-vaccination era were 3, 19F, 14, 6B, 6A, 19A, and 23F, with serotype 3 found exclusively in adults [23]. In the present study, the most notable decrease was observed in serotypes 19F and 14. While these changes were observed across all age groups, they were most pronounced among children under 5 years of age. The changes in other PCV10/PCV13 vac-cine types were less pronounced, with decreases in 6A, 6B, 23F, and 4 and a slight increase in 18C.

In the post-PCV period, invasive serotype 3 isolates expressed the most substantial increase, with a particularly sharp rise in children under 5 years of age. It is well known that the immunologic response induced by the conjugate vaccine to the serotype 3 polysaccharide appears to differ from the response induced by other vaccine serotypes, and it is often not satisfactory [24]. However, after comparing serotype 3 disease rates between countries that have introduced either PCV10, which does not include serotype 3, or PCV13 in their NIP, Sing et al. [25] reported that countries with a PCV10 NIP have shown a substantial linear increase in serotype 3 pneumococcal disease among all age groups since the introduction of the vaccine, while countries with a PCV13 NIP experienced a modest decline in serotype 3 incidence during the 3–4 years after vaccine introduction, followed by an upward trend in subsequent years. A notable increase was also observed in serotype 19A, with its frequency nearly doubling, and the most significant rise occurred in children under 5 years of age. Considering that PCV13 has been administered in Serbia only for the last two years, the observed change in serotype prevalence is predominantly attributable to the administration of PCV10, which has been in use during the preceding four years. Although a decline in PCV10/13 serotypes was observed across all age groups, the most significant changes occurred in the vaccine target population (children under 2 years of age). Similar to our findings, a rebound in the incidence of serotype 3 IPD among children <2 years, as well as those <5 years, was seen in France and Switzerland, respectively [26,27]. On the other hand, the coverage of higher valent PCVs—PCV13, PCV15, and PCV20—was only slightly reduced in the post-PCV period, especially in adults.

Researchers from Brazil [28] demonstrated that five years after the introduction of PCV10 into the NIP, there was a significant decline in VT-IPD, especially in children up to 2 years of age, where this change was observed as early as two years after the vaccine’s application. Additionally, the same authors in another paper declare that following PCV10 introduction, serotypes 3 and 19A are the most common in younger children, which is in line with our findings [29].

Furthermore, researchers from Finland did not observe an increase in serotypes 6A and 19A (PCV13/non-PCV10) three years following the introduction of PCV10 [30]. This lack of increase was attributed to cross-protection from serotypes 6B and 19F, which aligns with the findings of the current study. In a subsequent publication, authors from Finland stated that PCV10 results in long-lasting direct cross-protection against 6A. However, for 19A, they did not observe any reduction in serotype 19A six years after PCV10 introduction [31]. In 2018, the *Streptococcus pneumoniae* Invasive Disease Network (SpIDnet), established in 2012 by the European Centre for Disease Prevention and Control, reported an analysis of the impact of PCV on IPD in ten European countries over eight years of infant PCV programs using PCV10 and PCV13. The study found that the percentage of serotypes included in PCV13, PCV15, and PCV20 declined significantly over the study period [13]. Based on a comparison of serotype prevalence between 2012 and 2018, the authors concluded that PCV serotype coverage decreased in children under 5 years old in 2018 to 23% for PCV13, 32% for PCV15, and 63% for PCV20. Also, they noticed a difference between countries using PCV10 and those using PCV13 and found that the proportions of serotypes included in these three PCVs were greater in sites using PCV10 than in those using PCV13. In countries with universal PCV13 immunization, the prevalence of serotypes in children under 5 years old decreased in 2018 to 15% for PCV13, 26% for PCV15, and 63% for PCV20. In contrast, in countries using PCV10, the serotype coverage was higher: 43% for PCV13, 52% for PCV15, and 69% for PCV20. In Serbia, such significant changes have not yet occurred, as the coverage of PCV13, PCV15, and PCV20 in children under 2 years old remains relatively high in the post-PCV period, ranging from 63.9% for PCV13 to 70.5% for PCV20. Universal vaccination of children with PCV also has an indirect effect on the unvaccinated population, particularly benefiting those over 65 years of age, who experience a higher incidence of IPD compared to other age groups. The SpiDNet group claimed that changes in vaccine serotype IPD in older adults follow the same pattern of serotype changes in children with very limited delay but at a lower magnitude [32]. In our country, there has also been a decline in PCV coverage among adult patients. However, unlike European countries, where PCV13 serotype coverage among those over 65 was 36% in those using PCV10 and 20% in those with PCV13 immunization [13], Serbia maintained relatively high PCV serotype coverage after 2018: 72.3% for PCV13 and 86.1% for PCV20 in individuals ≥65 years. The degree of reduction in PCV serotype coverage in these countries was likely influenced by their previous use of PCV7. Therefore, it is expected that the serotype coverage in vaccines after several years of using PCV7/PCV10 or PCV7/PCV13 will be lower than in Serbia, where PCV7 was not previously used.

As anticipated, the decline in PCV10-VT serotypes was accompanied by a notable increase in non-PCV10 serotypes, primarily resulting in a gradual rise of serotypes 3 and 19A. Among non-PCV13 serotypes, there was an observed increase, in the following order of 9N, 10A, 22F, 15A,15B, 15C, 6C, 6D, and 7C across all isolates. These changes were most pronounced in the vaccine target group, namely children under 2 years old. In population older than 65 years, the dominance of serotype 22F among non-PCV13 strains has already been reported [28]. Indeed, serotype 22F is a very common PCV15/non-PCV13 serotype, while 15A and 6C are emerging as leading non-PCV20 serotypes in children under 7 years old [33,34]. The relatively recently discovered serotypes 6C and 6D are also increasing among our non-PCV isolates, particularly in children under 2 years and individuals over 65. This trend has also been observed in other countries in the post-PCV era [32,33]. It is important to note that our serotype 6C isolates are highly resistant to antibiotics, consistent with findings reported in the literature [35]. There is evidence that PCV13 vaccination consistently protects against 6C IPD in children [35]. Interestingly, the invasive serotype 6D was first reported in Europe by authors from Finland in 2011, shortly after the introduction of PCV10 in 2010 [36]. Additionally, numerous studies [33,34] reported that serotypes increasing in our study (10A, 9N, 15B, and 15C) have become very common in the post-PCV period. Serotype 8, which is dominant in many countries during the post-PCV period [37], actually declined twice in our country after 2018. In the post-PCV period, it is more frequently isolated in children aged 2–5 years old, whereas in the earlier period, it was found exclusively in adults.

Due to economic reasons, Serbia re-introduced PCV10 as free of charge in a NIP in 2024; bearing this in mind, it is crucial to carefully and closely monitor further changes in serotypes, especially considering the scenario observed in Belgium. Following the introduction of PCV13 in 2013, the switch to PCV10 in 2017 resulted in a resurgence of serotype 19A [38].

Unfortunately, due to inadequate reporting of IPD in Serbia, the true incidence of IPD remains unknown, and the impact of vaccines on the IPD rate cannot be effectively monitored. Based on voluntary passive laboratory surveillance of IPD conducted by the NRL for Streptococci, the notification rates varied throughout the study period. Several factors, in addition to vaccination, influenced these variations: the willingness of microbiologists to submit isolates to the NRL, antibiotic treatment prior to laboratory diagnosis, lack of molecular identification of IPD in some healthcare facilities, the effects of the COVID-19 pandemic and consequent insufficiency of the surveillance system. Therefore, the reported notification rates should not be used as indicators of IPD incidence, as they may lead to an underestimation of the actual rates.

As demonstrated in a previous report [23], the rate of resistance and reduced susceptibility to antibiotics among invasive pneumococcal isolates was high in Serbia. After the introduction of PCV, there was a significant decrease in antibiotic non-susceptibility among invasive pneumococcal isolates for almost all antibiotics. The incidence of non-susceptibility to penicillin and ceftriaxone among the isolates significantly decreased, while resistance to macrolides, despite a decrease, remained relatively high. Our results are in accordance with the results of a study in which the authors analyzed the results from 104 countries and identified reductions in the prevalence of non-susceptibility and resistance to penicillin and third-generation cephalosporins, while they did not find evidence of changes in the proportion of isolates non-susceptible to macrolides and tetracycline after PCV implementation. In addition to the impact of PCV in reducing resistance by lowering the incidence of highly resistant vaccine serotypes (19F, 14, 6A, 6B, 23F), the decrease in penicillin resistance can also be attributed to the reduced use of narrow-spectrum penicillins. In Serbia, a statistically significant decrease in the consumption of narrow-spectrum penicillins and first- and second-generation cephalosporins (ampicillin, amoxicillin, cefalexin, and cefaclor) was observed from 2017 to 2021. The persistence of relatively high macrolide resistance is most likely due to the extensive consumption of azithromycin during the COVID-19 period (2020–2022) in Serbia. [39]. Similar rates of non-susceptibility to penicillin and resistance to macrolides in invasive *S. pneumoniae* isolates have been reported in Spain, France, and Croatia. In contrast, significantly lower rates (<10%) have been observed in Finland, Norway, Sweden, Denmark, Germany, the Netherlands, and Slovenia. The lowest antibiotic susceptibility among pneumococci was found in Turkey and Romania, where resistance rates exceeded 35% [40].

As before 2018, the same serotypes (6A, 6B, 14, 19F, 19A, and 23F) continued to exhibit higher rates of resistance to one or more antibiotics, while serotype 3 remained significantly more sensitive than the others. The decline in antibiotic resistance can be attributed to the suppression of vaccine-resistant serotypes that were dominant before 2018 and the rise in susceptible serotype 3. Similar results have been reported in other countries. In Brazil, antimicrobial non-susceptible pneumococci decreased in the three years following PCV10 introduction; however, they subsequently increased and were associated with non-PCV10 types [28]. Considering the increase in the highly resistant serotype 19A in our country and its potential for further increase following the re-introduction of PCV10 in 2024, we can anticipate a renewed rise in pneumococcal resistance. Additionally, it is worrisome, given that serotype 19A is known for its ability to increase rapidly due to its high carriage prevalence, resistance rates, and invasiveness [38].

The comparison of the study period revealed significant shifts in ST and serotype distributions. Of the 78 STs identified, 44 were exclusive to the pre-PCV period, whereas 15 were unique to the post-PCV period. Notably, within the STs exclusive to the pre-PCV period, 31 were linked to PCV13 serotypes compared to 13 in the post-PCV period. This indicates that PCV13 has successfully reduced the prevalence of targeted serotypes, as observed in other countries [32,41]. The present study observed a significant decrease in serotypes 19F and 14. In the pre-PCV period, serotype 19F was detected among nine STs (ST63, ST179, ST191, ST271, ST320, ST416, ST2101, ST3897, and ST8144). After the introduction of PCVs, it was detected among four STs, of which three STs (ST450, ST8678, and new ST) were not detected in the pre-PCV period, and ST320 was detected only shortly after the introduction of PCV10 and was replaced completely with serotype 19A afterward. In the pre-PCV period, seven STs were associated with serotype 14 (ST15, ST15_gdh, ST143, ST156, ST550, ST3347, and ST18090), while in the post-PCV period, it was detected among only three STs (ST15, ST66, and ST143). Since two years of PCV13 administration is unlikely to significantly impact serotype distribution, the persistence of serotype 3 isolates among the circulating pneumococcal clones is not unexpected. This persistence can be attributed to several factors, including the suboptimal efficacy of PCVs against serotype 3 and its inherent virulence characteristics [42]. Additionally, the genetic plasticity of pneumococci allows serotype 3 to adapt and survive in a vaccinated population.

Potential capsule-switching events after introducing the PCVs were observed in ST66, ST230, ST242, ST320, and ST386. Some of these events likely represent pneumococcal adaptation mechanisms to evade vaccine-induced immunity. For example, the serotype switch from 19F to 19A within ST320 was observed previously in other countries after the introduction of PCV10 [43,44].

The limitation of this study is the relatively small number of isolates, especially in two age groups of the pediatric population, namely children from 2 to 5 years old and from 5 to 18 years old. Despite the study’s limitations, this is the first study demonstrating the overall impact of all PCVs used in Serbia on the population of invasive *S. pneumoniae.*

## 5. Conclusions

In conclusion, the introduction of PCVs has markedly influenced the epidemiology of specific pneumococcal STs and serotypes, leading to a substantial decrease in PCV10-targeted serotypes. Specifically, six years after the sequential introduction of PCVs in Serbia, there has been a noticeable decrease in serotypes 14, 19F, 6A, and 23F. The significant increase in serotype 19A, and particularly 3, during the post-vaccination period was remarkable. PCV10 vaccine coverage significantly dropped in the youngest. The introduction of PCV led to a decrease in resistance, particularly for first-choice antibiotics, beta-lactams, and macrolides, although macrolide resistance remained relatively high. Considering that PCV10 was re-introduced in our country in 2024, a further increase in serotype 19A and antibiotic resistance is expected. Therefore, it is essential to replace PCV10 with higher valent PCVs as soon as possible to address this trend effectively.

However, vigilant surveillance is critical to evaluating the burden of IPD and enabling the understanding of the broader impact on the epidemiology of IPD serotypes, which will help in developing the most effective prevention strategies and adaptive vaccine formulations.

## Figures and Tables

**Table 1:** Antibiotics non-susceptibility rate of the most common serotypes in the pre-vaccine and post-vaccine periods.

Serotype	Period (N)	PEN	CEF	ER	TET	CL	SXT	CHL	P+ER	MDR	XDR
**3**	2010–2017 (81)	9.9%	2.5%	4.9%	6.2%	3.7%	3.7%	1.2%	1.2%	1.2%	1.2%
	2018–2023 (110)	4.5%	0.0%	5.5%	10.9%	1.8%	0.9%	1.8%	0.0%	1.8%	0.0%
**6A**	2010–2017 (26)	80.8%	11.5%	76.9%	23.1%	19.2%	11.5%	0.0%	65.4%	19.2%	3.8%
	2018–2023 (6)	100.0%	16.7%	100.0%	50.0%	16.7%	0.0%	0.0%	100.0%	50.0%	0.0%
**6B**	2010–2017 (27)	96.3%	40.7%	70.4%	70.4%	66.7%	74.1%	44.4%	66.7%	29.6%	48.1%
	2018–2023 (18)	66.7%	5.5%	61.1%	55.5%	50.0%	50.0%	50.0%	50.0%	33.3%	33.3%
**14**	2010–2017 (50)	94.0%	30.0%	62.0%	34.0%	56.0%	90.0%	20.0%	58.0%	28.0%	34.0%
	2018–2023 (21)	76.2%	28.6%	57.1%	33.3%	52.4%	61.9%	9.5%	52.4%	33.3%	28.6%
**18C**	2010–2017 (15)	13.3%	0.0%	20.0%	6.7%	0.0%	33.3%	0.0%	0.0%	0.0%	0.0%
	2018–2023 (16)	25.0%	6.3%	50.0%	18.8%	6.3%	31.3%	0.0%	12.5%	12.5%	0.0%
**19A**	2010–2017 (19)	47.4%	21.1%	42.1%	42.1%	31.6%	47.4%	0.0%	31.6%	42.1%	10.5%
	2018–2023 (27)	63.0%	25.9%	51.9%	77.8%	44.4%	66.7%	0.0%	40.7%	37.0%	29.6%
**19F**	2010–2017 (59)	79.7%	47.5%	86.4%	83.1%	84.7%	62.7%	5.1%	74.6%	40.7%	45.8%
	2018–2023 (7)	57.1%	42.9%	57.1%	71.4%	57.1%	42.9%	0.0%	57.1%	14.3%	42.9%
**23F**	2010–2017 (21)	81.0%	33.3%	61.9%	71.4%	19.0%	52.4%	38.1%	57.1%	38.1%	33.3%
	2018–2023 (7)	14.3%	0.0%	0.0%	14.3%	0.0%	14.3%	0.0%	0.0%	14.3%	0.0%
**Other**	2010–2017 (129)	27.9%	5.4%	24.0%	24.0%	12.4%	22.5%	8.5%	16.3%	16.3%	4.7%
	2018–2023 (120)	25.8%	5.8%	27.5%	28.3%	15.8%	12.5%	5.8%	20.0%	20.0%	2.5%
**Total**	2010–2017 (434)	50.2%	18.2%	42.5%	35.6%	30.7%	38.2%	10.6%	34.9%	21.0%	17.5%
	2018–2023 (332)	28.9%	7.5%	28.3%	28.9%	17.8%	19.6%	6.0%	20.2%	16.9%	7.8%

PEN, penicillin; ER, erythromycin; C3 CEF, ceftriaxone; CL, clindamycin; TET, tetracycline; SXT, trimethoprim-sulfamethoxazole; CHL, chloramphenicol; P+ER, penicillin and erythromycin co-resistance; MDR, multidrug-resistant; XDR, extensive drug resistance.

## Data Availability

The original contributions presented in the study are included in the article/Supplementary Materials. Further inquiries can be directed to the corresponding author.

## Supplementary Materials

The following supporting information can be downloaded at: https://www.mdpi.com/article/10.3390/vaccines12080940/s1, Supplementary Table S1: Detected circulating clonal complexes among 246 invasive *Streptococcus pneumoniae* isolates from Serbia.

## Appendix A

Study group for laboratory surveillance of invasive pneumococcal diseases: Lazar Ranin (Clinical Hospital Centre Zemun, Belgrade, Serbia), Tatjana Kurucin (Clinical Center of Vojvodina, Novi Sad, Serbia), Anita Sente Žigmanović (General Hospital Subotica, Serbia), Deana Medić (Faculty of Medicine, University of Novi Sad, Novi Sad, Serbia; Institute of Public Health of Vojvodina, Novi Sad, Serbia), Zorica Vasiljević (Mother and Child Health Care Institute of Serbia “Dr. Vukan Cupic”, Belgrade, Serbia), Olivera Hadžić (Mother and Child Health Care Institute of Serbia “Dr. Vukan Cupic”, Belgrade, Serbia), Suzana Laban Nestorović (University Children’s Hospital, Belgrade, Serbia), Tamara Đorđević (University Children’s Hospital, Belgrade, Serbia), Tanja Tošić (University Clinical Center of Serbia, Belgrade, Serbia), Radmila Popović (Medical Center Valjevo, Serbia), Momčilo Đurić (Military Medical Academy, Belgrade, Serbia), Mirjana Hadnađev (Institute for Pulmonary Diseases of Vojvodina, Sremska Kamenica, Belgrade), Snežana Delić (Community health center Sombor, Serbia), Marina Stojković (Clinical Hospital Center “Dragiša Mišović-Dedinje”, Belgrade, Serbia), Nataša Nedeljkov Tešin (Community health center Kikinda, Serbia), Lidija Terzin (General Hospital “Dr Đorđe Joanović”, Zrenjanin, Serbia), Gordana Nikolić (General hospital Šabac, Serbia), Nataša Trifunović (Community health center Sremska Mitrovica, Serbia), Olivera Milićev (General hospital Vršac, Serbia), Ljiljana Pavlović (Institute of Public Health “Dr Milan Jovanovic Batut”, Belgrade, Serbia), Milena Branković (General hospital Užice, Serbia), Vesna Karanović (Community health center Kraljevo, Serbia), Snežana Antić (Institute of Public Health Niš, Serbia), Slađana Milovanović (Community health center Leskovac), Nataša Miljković (Clinical Hospital Centre Zvezdara, Belgrade, Serbia).

## References

[1] https://www.cdc.gov/pneumococcal/php/surveillance/index.html.

[2] https://iris.who.int/bitstream/handle/10665/376776/9789240093461-eng.pdf?sequence=1.

[3] https://www.cdc.gov/vaccines/vpd/pneumo/hcp/recommendations.html.

[4] Reyburn R., Tsatsaronis A., von Mollendorf C., Mulholland K., Russell F.M. (2023). Systematic Review on the Impact of the Pneumococcal Conjugate Vaccine Ten Valent (PCV10) or Thirteen Valent (PCV13) on All-Cause, Radiologically Confirmed and Severe Pneumonia Hospitalisation Rates and Pneumonia Mortality in Children 0–9 Years Old. J. Glob. Health.

[5] Waight P.A., Andrews N.J., Ladhani S.N., Sheppard C.L., Slack M.P.E., Miller E. (2015). Effect of the 13-Valent Pneumococcal Conjugate Vaccine on Invasive Pneumococcal Disease in England and Wales 4 Years after Its Introduction: An Observational Cohort Study. Lancet Infect. Dis..

[6] Schuck-Paim C., Taylor R.J., Alonso W.J., Weinberger D.M., Simonsen L. (2019). Effect of Pneumococcal Conjugate Vaccine Introduction on Childhood Pneumonia Mortality in Brazil: A Retrospective Observational Study. Lancet Glob. Health.

[7] Richter S.S., Diekema D.J., Heilmann K.P., Dohrn C.L., Riahi F., Doern G.V. (2014). Changes in Pneumococcal Serotypes and Antimicrobial Resistance after Introduction of the 13-Valent Conjugate Vaccine in the United States. Antimicrob. Agents Chemother..

[8] Tsaban G., Ben-Shimol S. (2017). Indirect (Herd) Protection, Following Pneumococcal Conjugated Vaccines Introduction: A Systematic Review of the Literature. Vaccine.

[9] Weil-Olivier C., van der Linden M., de Schutter I., Dagan R., Mantovani L. (2012). Prevention of Pneumococcal Diseases in the Post-Seven Valent Vaccine Era: A European Perspective. BMC Infect. Dis..

[10] Mackenzie G.A., Plumb I.D., Sambou S., Saha D., Uchendu U., Akinsola B., Ikumapayi U.N., Baldeh I., Usuf E., Touray K. (2012). Monitoring the Introduction of Pneumococcal Conjugate Vaccines into West Africa: Design and Implementation of a Population-Based Surveillance System. PLoS Med..

[11] https://www.batut.org.rs/index.php?category_id=140.

[12] https://www.paragraf.rs/propisi/pravilnik-o-programu-obavezne-i-preporucene-imunizacije-stanovnistva-protiv-odredjenih-zaraznih-bolesti.html.

[13] Hanquet G., Krizova P., Dalby T., Ladhani S.N., Nuorti J.P., Danis K., Mereckiene J., Knol M.J., Winje B.A., Ciruela P. (2022). Serotype Replacement after Introduction of 10-Valent and 13-Valent Pneumococcal Conjugate Vaccines in 10 Countries, Europe. Emerg. Infect. Dis..

[14] Gillespie S.H., Ullman C., Smith M.D., Emery V. (1994). Detection of Streptococcus pneumoniae in sputum samples by PCR. J. Clin. Microbiol..

[15] Gajic I., Kabic J., Kekic D., Jovicevic M., Milenkovic M., Mitic Culafic D., Trudic A., Ranin L., Opavski N. (2022). Antimicrobial Susceptibility Testing: A Comprehensive Review of Currently Used Methods. Antibiotics.

[16] The European Committee on Antimicrobial Susceptibility Testing Breakpoint Tables for Interpretation of MICs and Zone Diameters. Version 13.0. http://www.eucast.org.

[17] Mohanty S., Johnson K.D., Yu K.C., Watts J.A., Gupta V. (2022). A Multicenter Evaluation of Trends in Antimicrobial Resistance Among Streptococcus Pneumoniae Isolates From Adults in the United States. Open Forum Infect. Dis..

[18] Lee M.-C., Kuo K.-C., Lee C.-H., Hsieh Y.-C., Tsai M.-H., Huang C.-T., Huang Y.-C. (2020). The Antimicrobial Susceptibility in Adult Invasive Pneumococcal Disease in the Era of Pneumococcus Vaccination: A Hospital-Based Observational Study in Taiwan. J. Microbiol. Immunol. Infect..

[19] Cillóniz C., Rodríguez-Hurtado D., Torres A. (2018). Characteristics and Management of Community-Acquired Pneumonia in the Era of Global Aging. Med. Sci..

[20] Enright M.C., Spratt B.G. (1998). A Multilocus Sequence Typing Scheme for Streptococcus Pneumoniae: Identification of Clones Associated with Serious Invasive Disease. Microbiology.

[21] Francisco A.P., Vaz C., Monteiro P.T., Melo-Cristino J., Ramirez M., Carriço J.A. (2012). PHYLOViZ: Phylogenetic Inference and Data Visualization for Sequence Based Typing Methods. BMC Bioinform..

[22] Setchanova L., Alexandrova A., Pencheva D., Sirakov I., Mihova K., Kaneva R., Mitov I. (2018). Rise of Multidrug-Resistant Streptococcus Pneumoniae Clones Expressing Non-Vaccine Serotypes among Children Following Introduction of the 10-Valent Pneumococcal Conjugate Vaccine in Bulgaria. J. Glob. Antimicrob. Resist..

[23] Opavski N., Jovicevic M., Kabic J., Kekic D., Vasiljevic Z., Tosic T., Medic D., Laban S., Ranin L., Gajic I. (2023). Serotype Distribution, Antimicrobial Susceptibility and Molecular Epidemiology of Invasive Streptococcus Pneumoniae in the Nine-Year Period in Serbia. Front. Microbiol..

[24] Lapidot R., Shea K., Yildirim I., Cabral H., Pelton S., Massachusetts Department of Public Health (2020). Characteristics of Serotype 3 Invasive Pneumococcal Disease before and after Universal Childhood Immunization with PCV13 in Massachusetts. Pathogens.

[25] Sings H.L., Gessner B.D., Wasserman M.D., Jodar L. (2021). Pneumococcal Conjugate Vaccine Impact on Serotype 3: A Review of Surveillance Data. Infect. Dis. Ther..

[26] Ouldali N., Varon E., Levy C., Angoulvant F., Georges S., Ploy M.-C., Kempf M., Cremniter J., Cohen R., Bruhl D.L. (2021). Invasive Pneumococcal Disease Incidence in Children and Adults in France during the Pneumococcal Conjugate Vaccine Era: An Interrupted Time-Series Analysis of Data from a 17-Year National Prospective Surveillance Study. Lancet Infect. Dis..

[27] Oyewole O.R.-A., Lang P., Albrich W.C., Wissel K., Leib S.L., Casanova C., Hilty M. (2021). The Impact of Pneumococcal Conjugate Vaccine (PCV) Coverage Heterogeneities on the Changing Epidemiology of Invasive Pneumococcal Disease in Switzerland, 2005–2019. Microorganisms.

[28] Brandileone M.-C.C., Almeida S.C.G., Minamisava R., Andrade A.-L. (2018). Distribution of Invasive Streptococcus Pneumoniae Serotypes before and 5 Years after the Introduction of 10-Valent Pneumococcal Conjugate Vaccine in Brazil. Vaccine.

[29] Almeida S.C.G., Lo S.W., Hawkins P.A., Gladstone R.A., Cassiolato A.P., Klugman K.P., Breiman R.F., Bentley S.D., McGee L., Brandileone M.-C.d.C. (2021). Genomic Surveillance of Invasive Streptococcus Pneumoniae Isolates in the Period Pre-PCV10 and Post-PCV10 Introduction in Brazil. Microb. Genom..

[30] Jokinen J., Rinta-Kokko H., Siira L., Palmu A.A., Virtanen M.J., Nohynek H., Virolainen-Julkunen A., Toropainen M., Nuorti J.P. (2015). Impact of Ten-Valent Pneumococcal Conjugate Vaccination on Invasive Pneumococcal Disease in Finnish Children—A Population-Based Study. PLoS ONE.

[31] Rinta-Kokko H., Palmu A.A., Auranen K., Nuorti J.P., Toropainen M., Siira L., Virtanen M.J., Nohynek H., Jokinen J. (2018). Long-Term Impact of 10-Valent Pneumococcal Conjugate Vaccination on Invasive Pneumococcal Disease among Children in Finland. Vaccine.

[32] Savulescu C., Krizova P., Lepoutre A., Mereckiene J., Vestrheim D.F., Ciruela P., Ordobas M., Guevara M., McDonald E., Morfeldt E. (2017). Effect of High-Valency Pneumococcal Conjugate Vaccines on Invasive Pneumococcal Disease in Children in SpIDnet Countries: An Observational Multicentre Study. Lancet Respir. Med..

[33] Lepoutre A., Varon E., Georges S., Dorléans F., Janoir C., Gutmann L., Lévy-Bruhl D. (2015). Impact of the Pneumococcal Conjugate Vaccines on Invasive Pneumococcal Disease in France, 2001–2012. Vaccine.

[34] Skoczyńska A., Kuch A., Sadowy E., Waśko I., Markowska M., Ronkiewicz P., Matynia B., Bojarska A., Wasiak K., Gołębiewska A. (2015). Recent Trends in Epidemiology of Invasive Pneumococcal Disease in Poland. Eur. J. Clin. Microbiol. Infect. Dis..

[35] Grant L.R., Hanquet G., Sepúlveda-Pachón I.T., Theilacker C., Baay M., Slack M.P.E., Jodar L., Gessner B.D. (2024). Effects of PCV10 and PCV13 on Pneumococcal Serotype 6C Disease, Carriage, and Antimicrobial Resistance. Vaccine.

[36] Nahm M.H., Oliver M.B., Siira L., Kaijalainen T., Lambertsen L.M., Virolainen A. (2011). A Report of Streptococcus Pneumoniae Serotype 6D in Europe. J. Med. Microbiol..

[37] Tin Tin Htar M., Christopoulou D., Schmitt H.-J. (2015). Pneumococcal Serotype Evolution in Western Europe. BMC Infect. Dis..

[38] Desmet S., Peetermans W., Lagrou K. (2018). Switch in Childhood Pneumococcal Vaccine in Belgium. Lancet Infect. Dis..

[39] Medic D., Bozic Cvijan B., Bajcetic M. (2023). Impact of Antibiotic Consumption on Antimicrobial Resistance to Invasive Hospital Pathogens. Antibiotics.

[40] (2023). Anti-Microbial Resistance Surveillance in Europe 2023–2021 Data.

[41] Redin A., Ciruela P., de Sevilla M.F., Gomez-Bertomeu F., Gonzalez-Peris S., Benitez M.A., Trujillo G., Diaz A., Jou E., Izquierdo C. (2021). Serotypes and Clonal Composition of Streptococcus Pneumoniae Isolates Causing IPD in Children and Adults in Catalonia before 2013 to 2015 and after 2017 to 2019 Systematic Introduction of PCV13. Microbiol. Spectr..

[42] Cleary D.W., Lo S.W., Kumar N., Bentley S.D., Faust S.N., Clarke S.C. (2023). Comparative Genomic Epidemiology of Serotype 3 IPD and Carriage Isolates from Southampton, UK between 2005 and 2017. Microb. Genom..

[43] Mott M.P., Caierão J., Cunha G.R., Del Maschi M.M., Pizzutti K., D’Azevedo P., Dias C.A.G. (2019). Emergence of Serotype 19A Streptococcus Pneumoniae after PCV10 Associated with a ST320 in Adult Population, in Porto Alegre, Brazil. Epidemiol. Infect..

[44] Hsieh Y.-C., Lin T.-L., Chang K.-Y., Huang Y.-C., Chen C.-J., Lin T.-Y., Wang J.-T. (2013). Expansion and Evolution of Streptococcus Pneumoniae Serotype 19A ST320 Clone as Compared to Its Ancestral Clone, Taiwan19F-14 (ST236). J. Infect. Dis..

